# Absence of family support and associated factors in the urban births of a city in Southern Brazil: fact or fiction?

**DOI:** 10.1590/1980-549720230053.2

**Published:** 2023-12-01

**Authors:** Caroline Bender de Quadros, Mariana Bonati de Matos, Bárbara Borges Rubin, Jéssica Puchalski Trettim, Gabriela Kurz da Cunha, Carolina Coelho Scholl, Kênia Cordeiro Silva, Maria Eduarda Centena Duarte Vieira, Luciana de Avila Quevedo, Ricardo Tavares Pinheiro

**Affiliations:** I Universidade Católica de Pelotas, Programa de Pós-Graduação em Saúde e Comportamento – Pelotas (RS), Brasil.; II Universidade Federal de Pelotas, Programa de Pós-Graduação em Epidemiologia – Pelotas (RS), Brasil.

**Keywords:** Medical chaperones, Humanized delivery, Obstetric delivery, Birth, Acompanhantes formais em exames físicos, Parto humanizado, Parto obstétrico, Nascimento

## Abstract

**Objective:**

To verify the prevalence and identify the factors associated with the absence of birth companions among women in Southern Brazil.

**Methods:**

This is a cross-sectional study carried out with 466 parturient women in a cohort of women from the urban area of the city of Pelotas, RS. At 18 months postpartum, a structured questionnaire was applied with sociodemographic, gestational data and questions related to childbirth. Logistic regression was performed to adjust for possible confounding factors.

**Results:**

The prevalence of the absence of a birth companion among women was 22.3%. Parturient women with up to 8 schooling years (PR=2.0 [95%CI 1.1–3.8]), who did not live with a partner (PR=2.3 [95%CI 1.2–4.3]), who performed their prenatal care in the public sector (PR=1.9 [95%CI 1.0–3.7]) and who had a cesarean delivery (PR=6.0 [95%CI 2.9–12.4]) were more likely to not have had a birth companion.

**Conclusion:**

The results shows relevant evidence for the verification of the presence of a companion in Southern Brazil, indicating the need for better use and adherence to this practice. In addition, the law that approves the presence of the birth companion in Brazil does not seem to be being fully implemented, disrespecting a right of parturient women and impacting the benefits for for maternal and child health.

## RESUMO

**Objetivo:**

Verificar a prevalência e identificar os fatores associados à ausência do acompanhante de parto em mulheres no sul do Brasil.

**Métodos:**

Trata-se de um estudo transversal, realizado com 466 parturientes, pertencentes a uma coorte de mulheres da zona urbana da cidade de Pelotas, RS. Aos 18 meses pós-parto, foi aplicado um questionário estruturado com dados sociodemográficos, gestacionais e questões relacionadas ao parto. Foi realizada regressão logística para ajustes de possíveis fatores de confusão.

**Resultados:**

A prevalência da ausência de acompanhante de parto entre as mulheres foi de 22,3%. As parturientes com até 8 anos de estudo (RP=2,0 [IC95% 1,1–3,8]), que não viviam com um companheiro (RP=2,3 [IC95% 1,2–4,3]), que realizaram o pré-natal no setor público (RP=1,9 [IC95% 1,0–3,7]) e que tiveram um parto via cesárea (RP=6,0 [IC95% 2,9–12,4]) apresentaram maior probabilidade de ausência de acompanhante de parto.

**Conclusão:**

Os resultados apontam evidências relevantes para o seguimento da verificação da presença do acompanhante de parto no sul do Brasil, indicando a necessidade de melhor aproveitamento e adesão desta prática. Além disso, a lei que aprova a presença do acompanhante de parto no Brasil parece não estar sendo colocada em prática de modo integral, desrespeitando um direito das parturientes e impactando nos benefícios para a saúde materno-infantil.

## INTRODUCTION

Historically, the birth process was considered as a natural and social event, which traditionally occurred in the family group, with the assistance of midwives; the physician was called only in severe cases. After the XX century, the primary characteristics of birth were gradually replaced and began to take place in hospitals, with medical technicians, thus reducing mortality and morbidity rates; however, it led to the loss of autonomy for the parturient woman regarding her birth and the absence of family support^1-3^.

In order to promote a more favorable birth environment, that prioritizes the women’s safety and well-being, besides minimizing non-essential interventions, the World Health Organization (WHO) recommended guidelines that emphasize the centrality of mother and child in the care model. These recommendations aim at reaching more positive physical and psychological outcomes during the entire birth process. The approach proposed by the WHO emphasizes female autonomy, emotional support, adequate information, and personalization of care, contributing with a more respectful and satisfactory delivery. In this scenario, the use of non-pharmacological interventions (NPIs) and a birth companion are recommended^4^.

Continuous accompaniment during labor can provide emotional benefit (giving support and encouragement), physical support (assisting with baths, changing of positions, reduction of pain and massages), and intermediation between the wills of the parturient and health professionals^2^. The benefits of the presence of a birth companion also include the increasing number of spontaneous vaginal births, reduction of intrapartum analgesia, as well as the reduction of labor duration, c-sections and instrumental vaginal birth^2^. Besides, it has been associated with a more satisfying birth experience for the woman^5^ and better 5-minute Apgar score after normal delivery^6^.

In some countries, having a birth companion is a normal practice, and many hospitals provide the “Plan of Delivery or Birth”. In England, for instance, the Plan of Delivery has been offered since 1993, and the goal is to provide the parturient with the choice for the procedures she wishes to have in her delivery; among them, to have or not to have a companion of her choice, which could be the technical support of a doula and a member of the woman’s family network. In Canada, among the family members who accompany the woman during labor, the husband/partner has corresponded to 95% of the preference and, in France, 99%^7,8^.

In Portugal, where the right for a companion has been ensured by law since 1985, a national study with women showed that 54.5% of participants had a companion during birth. When referring to normal delivery, the prevalence increased to 74.5%^9^. In Brazil, Law . 11,108. Which guarantees the right to a companion during labor, delivery and immediate postpartum was only sanctioned in 2005^10,11^. However, data from the study *Nascer no Brasil,* which interviewed 23,940 of postpartum women in 2011 and 2012, indicated that 24.5% of the women had total absence of a birth companion and, considering the regions, the South presented 19.5% of absence^12^.

A study carried out in the South of Brazil showed that little more than half of the pregnant women was informed about the right to have a companion during labor, and less than 8.0% had a Plan of Delivery^5^. Another study, conducted in the Southeast, showed that even though 57.1% of the women were accompanied during labor, only 38.1% had a companion at moment of delivery^13^.

There are only few studies investigating the sociodemographic factors associated with the presence of a birth companion. Of the existing ones, *Nascer no Brasil* showed that c-sections, lower income and schooling, multiparity and being a user of the public sector presented higher chances of not having a birth companion at any point, from hospitalization to delivery^12^. Another study showed that the parturient woman’s schooling was not related to the presence of a companion during delivery, but instead, in the postpartum period. The proportion of women with a companion was higher among those with complete high school^14^.

Considering the importance of the subject and the lack of studies in the literature that investigate the factors associated with the absence of family support during birth in Brazil, this study aimed at verifying the prevalence of a birth companion and describing the factors associated with the absence of a companion among parturient women in the extreme South of Rio Grande do Sul (RS).

## METHODS

This is a cross-sectional study, nested in a cohort called “Maternal neuropsychiatric disorders in the pregnancy-puerperal cycle: detection and early intervention and its consequences in the family triad”. The cohort study to which this analysis is related has followed-up women from the gestational to the postpartum period and includes several phases of accompaniment.

The sample was collected from 2016 to 2018, through the listing of 488 census sectors that compose the urban zone of the city of Pelotas (RS), according to the census mesh block of 2010, of the Brazilian Institute of Geography and Statistics (IBGE)^15^, as primary sample units. Then, a random sample was performed, and 244 sectors were selected (50% of the total). Each one of the selected sectors was visited by a team that, through an active search, visited all residences in the selected sector searching for pregnant women who met the inclusion criteria: being up to 24 weeks pregnant and living in one of the selected sectors.

A sample calculation for the cohort study was performed to estimate 80% power, based on the parameters of the 20% post-partum depression prevalence (main objective of the cohort study). With a 30% increase for losses and refusals, 514 pregnant women would be required. However, considering other objectives related to the cohort study, such as this analysis, a higher number of pregnant women was necessary. More details about the cohort methodology can be accessed in the publications of Pinheiro et al.^16,17^ The evaluation of accompaniment took place in four stages: the first one included 981 pregnant women between the first and the second gestational trimester (T1 — baseline), and was performed in the households of participants; the second one included 840 pregnant women and took place in the university hospital between sixty and ninety days after the first one (T2); the third one included 756 women and her children ninety days after delivery (T3); and the fourth one included 466 women (47.5% of the baseline) and her children 18 months after delivery (T4). It is worth to mention that the number of participants assessed in the fourth stage (T4) was owed to the interruption in data collection due to the COVID-19 pandemic. The two last stages of evaluation were performed in a room inside the university. The data in this study refer to the last stage of the evaluation (T4). The data were collected through printed surveys, by students in the health field who underwent previous training.

The economic class of the participants was evaluated using the classification of the Brazilian Association of Population Studies (ABEP), which is based on the accumulation of material goods and the schooling of the head of the family. This classification groups individuals in classes (A, B, C, D or E), considering that letter “A” refers to the highest economic class, and “E”, to the lowest economic class. For this study, the sample was classified in the following groups: A+B (high classes), C (middle class) and D+E (low classes))^18^. The economic class was considered as an exposure variable.

A general survey was also Applied and the following exposure variables: living with a partner (no, yes); schooling years (up to eight years, nine years or more); type of delivery (vaginal, c-section), prenatal location (private health insurance, public); experience of having a birth companion (it really helped delivery to be better and calmer; it helped delivery to be better and calmer a little; indifferent; it did not help, because it made me more nervous); the birth companion was the person of her choice (no, yes); who the birth companion was (child’s father, mother, aunt/godmother, others); and for parturient women who did not have a companion, if she would like to have had one (no, yes), and how they felt not having one (very bad, indifferent, good — but would have felt better with a companion, very well — I chose not to have a companion). It is important to mention that all variables were assessed through a close-ended question asked by the interviewer, with the aforementioned options of answers. The outcome variable was also part of the general questionnaire and was assessed through the following question: “Did you have a birth companion?”, and the answer could be “yes” and “no”.

The data were encoded and doubly typed in the Epi-Data software for consistency checking. Regarding data analysis, at first the univariate analysis was performed to obtain descriptive results through simple and relative frequencies. The bivariate analysis was conducted using the χ^2^ test. The multivariate analysis was performed using logistic regression. Multicollinearity between exposure variables was analyzed through the tolerance detection test. Values below 0.1 were considered as the existence of collinearity between variables. The economic class variable, for being colinear to the schooling variable, was not taken for multivariate analysis.

All participants signed the Informed Consent Term, and the project was approved by the Research Ethics Committee of the university, protocol number 47807915.4.0000.5339.

## RESULTS

**Figure 1.**
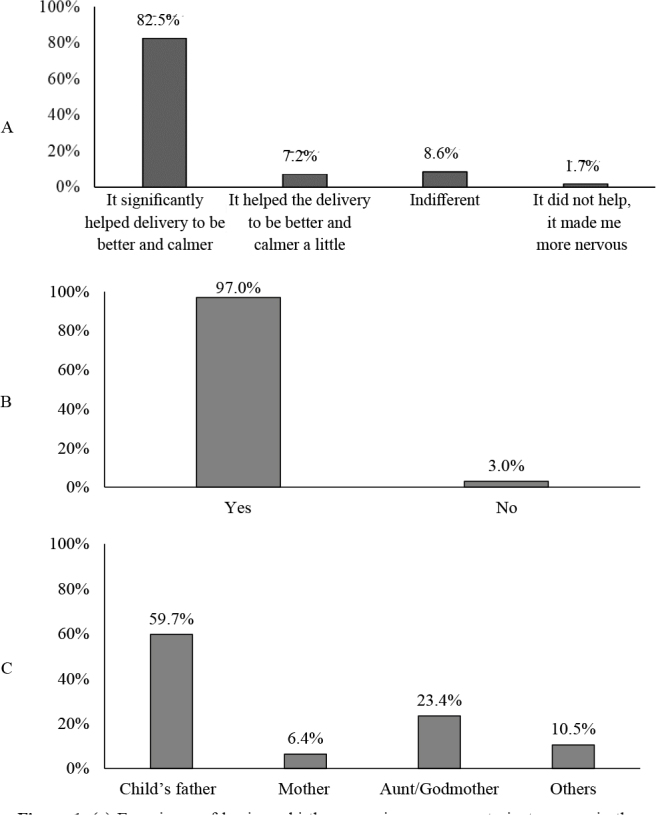
(a) Experience of having a birth companion among parturient women in the city of Pelotas, Brazil, 2018–2020; (b) Prevalence of a birth companion having been the one of choice among parturient women in the city of Pelotas, Brazil, 2018–2020; (c) Prevalence of kinship of the birth companion among parturient women in the city of Pelotas, Brazil, 2018–2020.

The sample was comprised of 466 women. The results showed that 77.7% (n=362) of parturient women had a birth companion. Of these, 82.5% (n=299) reported that the presence of this person contributed with a better and calmer delivery, 97.0% (n=351) had the companion of their choice, and 59.7% (n=278) chose the child’s father as their companion (Figure 1).

**Figure 2.**
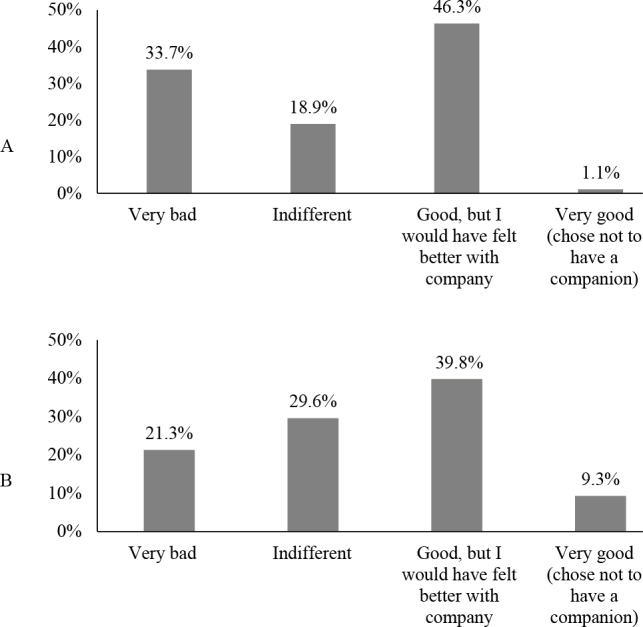
(a) Feeling of parturient women for not having had a birth companion in the city of Pelotas, Brazil, 2018–2020; (b) Current feeling of parturient women for not having had a birth companion in the city of Pelotas, Brazil, 2018–2020.

Parturient women who did not have a birth companion represented 22.3% (n=104) of the sample. Of these, 87.6% mentioned the desire to have had company. When asked about the feeling on the birth day, caused by not having company, 46.3% (n=44) mentioned feeling good, but they would have felt better with company. Regarding the fact of not having company, after knowing it was their right, 39.8% (n=43) of the women informed they felt good, but would have felt better if the right to the presence of a companion had been respected (Figure 2).

Table 1 presents the prevalence of the absence of a birth companion and the associated factors. According to the results, women belonging to economic classes D+E (p<0.001), who did not live with a partner (p=0.049), with lower schooling (p=0.002), who had a c-section (p<0.001) and who attended prenatal care at a public location (p=0.004), presented higher prevalence of absence of a birth companion.

**Table 1.** Prevalence of the absence of a birth companion and associated factors in the city of Pelotas (RS), Brazil, 2018–2020.

Variables	n (%)	Absence of a birth companion n (%)	p-value
Economic class			
High class (A+B)	123 (26.4)	18 (14.6)	<0.001
Middle class (C)	280 (60.1)	60 (21.4)	
Low class (D+E)	63 (13.5)	26 (41.3)	
Living with a partner			
No	76 (16.3)	24 (31.6)	0.049
Yes	390 (83.7)	80 (20.5)	
Schooling years*			
Up to 8	118 (25.4)	39 (33.1)	0.002
9 or more	347 (74.6)	65 (18.7)	
Type of delivery*			
Normal	132 (32.4)	11 (8.3)	<0.001
C-section	275 (67.6)	84 (30.5)	
Prenatal location*			
Private/health insurance	136 (31.4)	18 (13.2)	0.004
Public service	297 (68.6)	76 (25.6)	

* Variables with missing data.

Table 2 presents the final hierarchic model for the absence of company during birth among parturient women. The absence of a birth companion was associated to the variables: schooling (PR=2.0; 95%CI 1.1–3,8), living with a partner (PR=2.3; 95%CI 1.2–4.3), prenatal location (PR=1.9; 95%CI 1.0–3.7) and type of delivery (PR=6.0; 95%CI 2.9–12.4).

**Table 2.** Final hierarchical model for the absence of a birth companion among parturient women in the city of Pelotas (RS), Brazil, 2018–2020.

Variables	Prevalence ratio (PR)	95%CI
**1st level**		
Schooling years		
Up to 8	2.0	1.1–3.8
9 or more	Reference	
Living with a partner		
No	2.3	1.2–4.3
Yes	Reference	
**2nd level**		
Prenatal location		
Private/health insurance	Reference	
Public service	1.9	1.0–3.7
**3rd level**		
Type of delivery		
Normal	Reference	
C-section	6.0	2.9–12.4

95%CI: 95% confidence interval

Therefore, parturient women with up to eight schooling years had 2.0 (95%CI 1.1–3.8) more chances of not having a birth companion when compared to women with higher schooling. Women who did not live with a partner presented 2.3 (95%CI 1.2–4.3) more chances of not having a companion when compared to those who lived with a partner. Also, women who underwent prenatal care in the public sector had 1.9 (95%CI 1.0–3.7) more chances of not having a birth companion when compared to women who underwent prenatal care in the private sector. Finally, parturient women who had a cesarean section presented a proportion of 6.0 (95%CI 2.9–12.4) more chances of not having a birth companion when compared to women who had a normal delivery.

## DISCUSSION

This study aimed at verifying the prevalence of a birth companion and describing the factors associated with the absence of company among parturient women in the extreme South of Rio Grande do Sul (RS). The main findings show that parturient women with up to eight schooling years, who did not live with a partner, who underwent prenatal care in the public sector and who had a cesarean section had higher chances of not having a birth companion.

The prevalence of the absence of a birth companion was relatively high (22.3%) when compared to a similar study carried out in the capital of the state of Rio Grande do Sul (Porto Alegre, RS), with 385 women, in which the absence of company was of 9.4%^3^. In the research “*Nascer no Brasil*”, the absence of company was of 24.5%, and when related only to the South region, this level decreased to 19.5%^3,12^. Despite the approval of the national law that has officialized the permission of a birth companion since 2005, the presence of such a person is not totally allowed or encouraged in health institutions, as our findings show. This can be observed in a study in which more than one third of the pregnant women reported not having been informed about the possibility of having company at the time of delivery^5^. Even though it has been legal for almost two decades, not all health professionals are aware of the legislation that guarantees this right to pregnant women^19^.

Regarding socioeconomic characteristics, it was possible to observe that those with up to eight schooling years were more prone to the absence of a birth company. The study “*Nascer no Brasil*” is in accordance with the found data, since parturient women who did not have a birth companion belonged to social classes D+E and had lower schooling^12,20^. These data lead us to observe that women and their companions who presented lower schooling levels can be more vulnerable regarding the manifestation of a wish to have a birth companion, once they have less information regarding health rights during prenatal care^21^. Besides, it is possible that women with lower schooling have more difficulty to understand the guidelines and information provided by the health team. Therefore, it is essential that health professionals be capable of communicating clearly and efficiently so that this information can be assimilated by all schooling levels.

Besides, parturient women who did not live with a partner had lower chances of absence of a birth companion. The presence of a companion during the entire pregnant-puerperal cycle is extremely important for the mother-infant dyad, and has been related to protective factors, such as reduced infant mortality and better maternal health^22^. The presence of a partner during pregnancy and at the time of delivery may have repercussions in the quality of care received by the mother and infant after birth, reducing risks and contributing with maternal and child health^3^. However, when the pregnant woman has no partner, the health team should advise her to search for the presence of a relative or friend who can accompany her, once the birth companion can be anyone trusted by the woman.

When the use of the public and private network for prenatal care is analyzed, it was observed that parturient women who used the public system had higher chances of not having a birth companion, corroborating with the literature^12^. Among the justifications for this scenario in the public sector, infrastructure, lack of support from administration, busy institutional routine and prevalence of medical and staff willingness are often mentioned^21,23^. A study carried out in the state of Santa Catarina showed that the main difficulties to insert a companion regard the inadequate physical space and medical disapproval, once the health team does not consider the delivery and surgery room as a place for a companion, besides questioning the psychological preparation of the birth companion^24^.

Another important characteristic was the higher absence of a birth companion in c-sections than in normal deliveries. Also, the rate of absence of a companion in relation to the cesarean section found in this study is high, considering that when compared to a national study, the percentage of absence of a companion in c-sections remained lower in comparison to normal deliveries^12^. This date is also observed in a regional study, in which the presence of a birth companion among the three states of the South region and type of delivery become close (cesarean section, 34.8%; and vaginal delivery, 39.4%)^20^. This result can be explained by the mistaken explanation of health professionals about the permission of company only in cesarean sections. This claim is based on the belief that this type of birth is more controlled, and that in vaginal births other people would “disturb” the team^25^. However, the literature shows that the benefits of having a birth companion include the increase in spontaneous vaginal deliveries, as well as reduced time of labor, intrapartum analgesia and instrumental vaginal birth^2^.

Among the choices of parturient women for the companion, the child’s father was the most frequent one (76.4%), so enabling the early connection between father and infant from the start, leading to stronger family connection, creating confidence and safety to the mother^26,27^. In other studies, the choice of the child’s father was also prevalent, both nationally (35.4)^3,12^. For parturient women who had company, most reported that their presence helped significantly to maintain their calm during the labor processes. The data corroborate those of the study “*Nascer no Brasil*”, which resulted in 84.5% referring to the mentioned report^12^.

The presented results should be interpreted considering some limitations. Once this is a cross-sectional study, it is important to highlight the possibility of reverse causality. Additionally, it is relevant to weight on the potential presence of information bias, once the data in this study refer to the stages of pregnancy and delivery, being collected eighteen months after the event. It is also important to notice that the scientific literature suggests that other variables that were not approached in this study may influence the presence of a companion during delivery, such as skin color and history of previous deliveries (parity). Finally, despite the advantage of this study counting on a population base sample, which is a strong aspect, it is necessary to consider that the data may not completely reflect the geographic and cultural diversity of the Brazilian population, given the broad territory of our country. However, it is important to emphasize that, given the lack of studies about the sociodemographic factors associated with the presence of companions during labor, this study plays a scientifically relevant role in the construction of knowledge about the theme.

The implementation of birth companions in health institutions, besides being a right guaranteed for women, helps to meet the good practices indicated by the WHO^4^. Besides, it collaborates with the increase of spontaneous vaginal deliveries, reduction of intrapartum analgesia, duration of labor, c-sections, instrumental vaginal births, and brings more satisfaction to women regarding the birth experience^6^. To guarantee the presence of a companion chosen freely by the Woman, it is essential that, since prenatal care, there are actions of education and promotion of health so that pregnant women be informed about such a right. Therefore, it is up to the institutions in charge to provide training and development to health professionals about the related legislation, as well as the need for accessible information for all audiences.

To complement this study, it would be interesting to analyze how the right to a birth companion is informed during prenatal care appointments, especially in the public network, and the possibility to verify the justifications of health institutions about the absence of company. However, this study provided the visualization of the factors associated with the absence of a companion in the extreme South of Brazil, besides informing the participants about the right to have one. Therefore, the results presented here are relevant for the follow-up on the verification of the presence of a birth companion in the South of Brazil, indicating the need for better use and more adherence to the practice so that all women can use their benefits.
